# Protein–Phospholipid Interaction Motifs: A Focus on Phosphatidic Acid

**DOI:** 10.3390/biom8020020

**Published:** 2018-04-23

**Authors:** Emeline Tanguy, Nawal Kassas, Nicolas Vitale

**Affiliations:** Institut des Neurosciences Cellulaires et Intégratives, CNRS UPR3212 and Université de Strasbourg, 67000 Strasbourg, France; emeline.tanguy@etu.unistra.fr (E.T.); Nawal.Kassas@hotmail.com (N.K.)

**Keywords:** interaction motif, lipid binding, membrane, phospholipase D, phosphatidic acid

## Abstract

Cellular membranes are composed of thousands of different lipids usually maintained within a narrow range of concentrations. In addition to their well-known structural and metabolic roles, signaling functions for many lipids have also emerged over the last two decades. The latter largely depend on the ability of particular classes of lipids to interact specifically with a great variety of proteins and to regulate their localization and activity. Among these lipids, phosphatidic acid (PA) plays a unique role in a large repertoire of cellular activities, most likely in relation to its unique biophysical properties. However, until recently, only incomplete information was available to model the interaction between PA and its protein partners. The development of new liposome-based assays as well as molecular dynamic simulation are now providing novel information. We will review the different factors that have shown to modulate the capacity of PA to interact with specific domains in target proteins.

## 1. Introduction

The creation of a cell membrane to isolate the intracellular cytoplasm from the extracellular medium represented a major event in evolution. The so-called plasma membrane plays a critical role as a selective barrier, preventing the free diffusion of numerous small molecules such as ions, nucleotides, and diverse other biomolecules. This lipid interface also allows cells to maintain homeostasis by preventing the diffusion of important compounds needed for cell survival. Moreover, proteins transiently attached to or more directly embedded in the plasma membrane perform many vital cellular functions, such as transportation of molecules or organelles, signaling, and membrane reorganization. The importance of this class of proteins can be deduced by their relative prominence, as they form up to 40% of all cell proteins [1]. Among the class of proteins transiently associated with membranes, we can identify interactions based either on transient surface interaction or, alternatively, through post-translational modifications such as addition of a lipid to amino acids, allowing membrane anchoring. Most of the important biological functions involving signaling activity, membrane traffic, and transport rely on these membrane-associated proteins through their ability to transiently shuttle at the interface between the cytosol and lipid bilayers [2]. Modulation of the activity of peripheral membrane proteins often occurs after conformational changes resulting from membrane binding [2,3]. Alternatively, interactions with specific classes of lipids have also been reported to modulate directly the activity of transmembrane proteins such as ion channels or enzymes involved in lipid metabolism [2,4]. Understanding the mechanisms by which these proteins recognize and bind to their lipid partners represents a real challenge that would provide insights into the function of these proteins and might also facilitate future development of drugs to treat various pathological conditions.

Phosphatidic acid (PA) is one of the simplest glycerophospholipids and is present in only very small amounts in membranes (usually around 1%). Nevertheless, PA plays a key structural role as a precursor of most glycerophospholipids and has also been proposed to act as an important player in the transmission, amplification, and regulation of a great number of intracellular signaling and cellular functions [5]. Chemically, PA is comprised of a glycerol backbone to which are attached, through esterification, two fatty acyl chains and a phosphate at positions 1, 2, and 3, respectively. The unique feature of PA compared to the other diacyl–glycerophospholipids is its phosphomonoester link to a small anionic phosphate headgroup. At the molecular level, PA can interact with various proteins to modulate their catalytic activity and/or their membrane association, including guanosine triphosphatases (GTPases), kinases, phosphatases, nucleotide-binding proteins, and phospholipases [6]. Although a handful of proteins that present the capacity to bind to PA have been described, at least in the minimal lipid-strip assay, specific properties underlying protein–PA binding are still poorly understood. The particular phosphomonoester headgroup of PA, positioned close to the headgroup-acyl chain interface, is most likely critical in the binding of peripheral and transmembrane proteins. In agreement with the physiological importance of PA–protein interactions, many proteins have evolved domains that are relatively specific in their binding to PA [7]. In this article, rather than discussing the biological impact of protein–PA interactions that have been reviewed elsewhere [5,7,8], we will discuss the different biophysical parameters both for PA and proteins that contribute to effective and selective protein–PA interaction.

## 2. Phosphatidic Acid-Binding Modules

**Figure 1:**
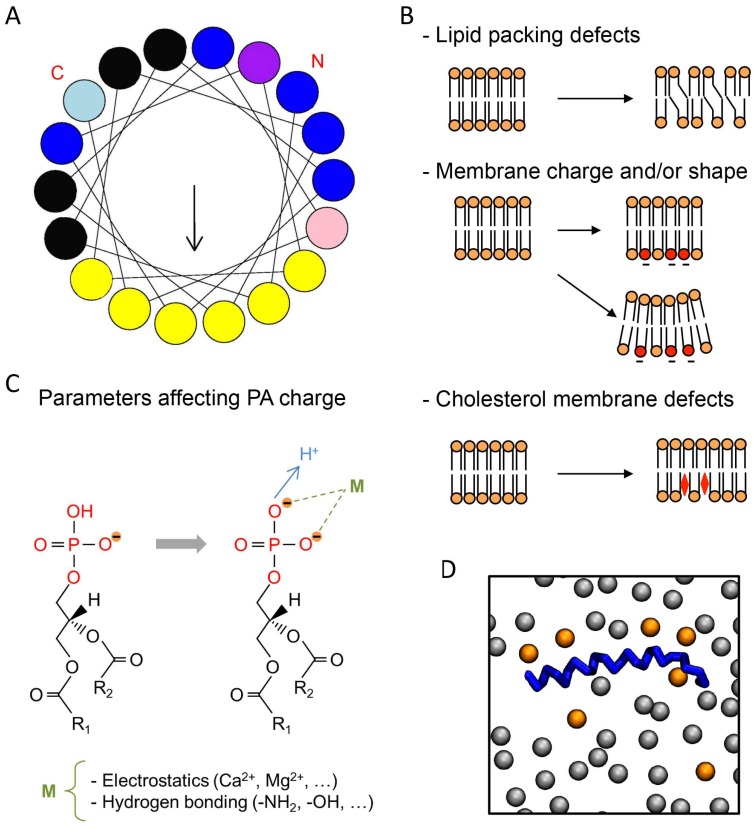
Typical phosphatidic acid (PA)-binding domain (PABD) and parameters affecting protein–PA interactions. (**A**) Amphipathic α-helix projection of a minimal characteristic PABD obtained with Heliquest software (http://heliquest.ipmc.cnrs.fr). Arrow indicates hydrophobic moment. Critical basic and hydrophobic residues are indicated in blue and yellow, respectively. Histidine (light blue), tryptophan (pink), and serine (purple) residues are also often present in the PABD. The amino-terminal (N) and carboxyl-terminal (C) regions of the PABD are indicated. (**B**) Different parameters affecting protein–PA binding are indicated on the right, including, from top to bottom: lipid-packing defects caused by fatty acyl unsaturation, negatively charged phospholipids such as phosphatidylserine (PS) (red) and membrane curvature, and cholesterol membrane defects (red diamond). Note that any of these parameters can affect PA binding either individually or in combination. Although cholesterol affects the hydrophobic core of the membrane or its hydrophilic interface, it is likely that the most important effect of cholesterol regarding the interaction of proteins with PA involves protein accessibility to hydrophilic headgroups of the membrane. (**C**) The net charge of PA is also modulated by different parameters such as ionic concentration and pH (adapted from [15]). The fatty acyl chain length and degree of unsaturation in the sn-1 or sn-2 positions (R_1_ and R_2_) also modify the binding to PABDs. (**D**) Top view of the final shot of a molecular dynamics simulation (500 ns) of the PABD of Spo20p (blue) in a phosphatidylcholine (PC)/PA (grey/orange) artificial membrane [16]. Only the phosphate headgroups of the lipids of a single bilayer are shown.

Using different in vitro assays, PA has been shown to interact with or regulate at least 50 different partners present in most organisms ranging from yeasts and plants to mammals [8]. The identification of PA-binding domains (PABDs) within PA effectors has proven to be challenging because it is not always clear whether protein–lipid interactions are specific for PA or whether they reflect an overall affinity for negatively charged lipids. Nevertheless, the idea that a short stretch of positively charged residues represents a major feature related to PA binding is emerging [8]. In agreement with this model, point mutations of basic residues within PABDs have often been found to reduce PA binding [7]. The current model proposes that a well-defined PA-recognition structural motif is unlikely to exist, but that a combination of positively charged and surface-exposed hydrophobic residues is responsible for the interaction with PA (Figure 1A). It is also clear that in addition to the charged lysine and arginine, certain amino acid residues like histidine, serine, and tryptophan are often found in PABDs (Figure 1A), but their exact function remains elusive.

Recently, a growing number of PABDs fused to fluorescent proteins, such as green fluorescent protein (GFP), have been used as probes to image dynamics of PA pools in living cells or to gain insight into the localization and function of PA partners. For instance, probes derived from the PABD of the yeast sporulation protein Spo20p, and the mammalian proto-oncogene Raf1 kinase, are among the most widely used. Intriguingly, when fused to GFP and overexpressed in cells, different PABDs are often found in different subcellular compartments. For instance, the PABD of Spo20p (Spo20p–PABD) generally accumulates in the nucleus or at the plasma membrane in mammalian cells [9,10,11], whereas the PABD of the yeast protein Opi1p (Opi1p–PABD) has the capacity to shuttle between the endoplasmic reticulum (ER) and the nucleus [12]. The PABD of the mammalian protein PDE4A1 (PDE4A1–PABD) is often associated with the Golgi apparatus [13]. Recent work excluded cell-specific expression or protein–protein interactions as causes for this differential localization. On the contrary, some evidence suggests that these different PABDs sense different pools of PA, each within a particular specific environment [14]. Among the in vitro assays used to study PA–protein interactions, lipid overlays—whereby single lipids are spotted from organic solution onto membranes—are widely used as an initial approach to study protein–lipid interaction. However, lipid overlay fails to present lipids in a bona fide membrane bilayer structure, thus making it untrustworthy. This is especially the case for PA, since (i) its protein recognition motif has been postulated to be rather undefined, and (ii) its head group moiety, unlike most phospholipids, is probably mostly buried within the hydrophilic region of the membrane. Therefore, for PA, liposome-binding assays mimicking the membrane bilayer environment are now more commonly used, as they allow us to replicate most membrane parameters, can provide complex lipid composition, and modulate several biophysical characteristics of bilayer membranes.

## 3. Effect of Membrane Topology and Environment on Phosphatidic Acid Binding

Several groups have recently shown that the ability of PABDs to bind PA is strongly affected by membrane lipid composition. Among the different factors that define the biophysical properties of membrane bilayers, membrane packing is important for protein–lipid interactions. Indeed, lipid composition in general, and the degree of unsaturation of the fatty acyl chain in phospholipids in particular, produces small defects in the geometrical arrangement of lipids. In other words, the generation of small spaces between the lipid head groups is proposed to favor the binding of certain peripheral proteins (Figure 1B). Indeed, it is presumed that small differences in the overall shape of cylindrical phospholipids caused by unsaturated fatty acids in the sn-2 position could create surface cavities that would favor insertion of polypeptides usually harboring an α-helical conformation. It is important to remember that most, if not all, PA-binding modules share an amphipathic α-helix with positively charged amino acids on one side and hydrophobic residues on the other (Figure 1A). In line with this observation, a point mutation altering the α-helix of Spo20p was shown to reduce PA binding [9,10]. Using the PABDs of the yeast proteins Spo20p and Opi1p and the mammalian protein PDE4A1, in a fluorescence-based liposome assay, we have recently shown that although the three PABDs are sensitive to global packing defects, the latter two are more sensitive than Spo20p–PABD [14]. In line with increased binding of these sensors to PA when PA headgroups are more freely available, we also found that reducing liposome diameter, thereby increasing curvature, also increased PABD binding [14]. Using a different liposome-based assay, the Kooijman group made similar observations testing a larger set of PABDs [17]. These findings may be correlated to specific differences found in terms of packing defects and spontaneous curvature of membranes within the diverse intracellular organelles, and the specific subcellular distribution of these PABDs when expressed as PA sensors fused to GFP [14].

Like phosphatidylcholine (PC), the phospholipid phosphatidylethanolamine (PE) is also zwitterionic, but contains a much smaller headgroup, both in terms of actual size and hydration, and as such is supposed to reduce headgroup packing. As a consequence, PE was initially postulated to facilitate protein binding to membrane in general via increased insertion of amphipathic protein domains into lipid membranes [18]. However, in addition to these small changes in the global packing of membranes, PE also modulates the charge of phosphomonoester-containing membrane lipids. Indeed, it was shown that the interaction of the PA phosphomonoester headgroup with the primary amine in the headgroup of PE causes an increase in the negative charge of the phosphate ([18] and see below). In summary, PE has potentially two distinct effects on membranes that could influence protein–PA binding: (i) creating negative curvature stress and (ii) increasing the negative charge of PA from −1 to −2 via the electrostatic–hydrogen bond switch mechanism. In agreement with the idea that these two parameters are important for protein binding to PA, PE generally facilitates PA binding when included in liposomes [17]. Furthermore, when charges were kept constant but positive curvature was created by simultaneous insertion of lysophosphatidylcholine (LPC), PE was less effective at promoting PA binding, highlighting the importance of membrane curvature stress for proteins to bind PA [17].

Changing cholesterol levels is another way to modulate lipid packing in membranes (Figure 1B), reflecting the diversity of biological membranes where cholesterol levels increase along the secretory pathway from low levels in the ER to high levels at the plasma membrane. We have found that higher levels of cholesterol enhance the binding of PA-containing liposomes to Spo20p–PABD and less so to PDE4A1–PABD [14]. On the contrary, PA binding to Opi1p–PABD was negatively affected by cholesterol [14]. Altogether, these findings indicate that local membrane topology has a profound effect on PA binding for proteins, and moreover that PABDs display differential sensitivity to this parameter.

## 4. Effect of Membrane Charge on Phosphatidic Acid Binding

One of the most significant determining factors for biomembrane structure and function is the presence of negative charges. In agreement, it is well recognized that the negative charges carried by anionic lipids in most biological membranes represent critical sites of attraction for positively charged (carrying basic amino acids) protein domains (Figure 1B). In liposome reconstitution assays, as in cellular membranes, the effects of electrostatic interaction can be quite effective, and they therefore represent useful assays to investigate the effect of this aspect on PA–protein interaction. When binding to lipids occurs through a cryptic stretch of basic amino acids, which like Velcro binds strongly beyond a certain level of negative charges, supplementary negative charges provided by other anionic lipids could also promote protein–lipid interactions. To directly test the action of the net charge on the capacity of distinct PABDs to bind to PA, increasing amounts of phosphatidylserine (PS) were added to the liposomes. This resulted in increasing charge in PA-containing liposomes and gradually enhanced binding to Spo20p–PABD [14,19] and PDE4A1–PABD [14], without significant modification in binding to Opi1p–PABD [14], once again revealing that various PABDs respond differently to this parameter. Rather intriguingly, different members of the Lipin PA-phosphatase family bind PA with varying affinity, probably because the number and specific arrangement of positive amino acids within their PABDs are quite different. It is therefore possible that different isoforms of Lipin present different sensitivities to the overall charge in membranes. Furthermore, the ability of Lipin-1 to bind PA also appears to be sensitive to phosphorylation, which is not the case for Lipin-3 [20]. The latter observation suggests that phosphorylation within or in proximity to the PABD could represent another mechanism by which PA binding is regulated.

Divalent ions such as calcium can also interact electrostatically with the negative charge formed by proton dissociation, hence altering the proton dissociation equilibrium and in consequence directly affect the amount of negative charges available for interaction (Figure 1C). Checking their action represents another way to probe the existence of physiologically relevant mechanisms to modulate protein–PA binding [21]. Micromolar concentrations of free calcium increase binding of Spo20p–PABD and PDE4A1–PABD to PA-containing liposomes. On the contrary, at physiological concentrations, calcium did not affect PA binding to Opi1p–PABD [14], in agreement with differences in calcium sensitivity between the different PABDs. The effect of membrane charge on protein–PA interaction can be summarized as follows: charge increase promotes electrostatic attraction between cationic amino acids in proteins containing PABD and thereby strengthens binding. In agreement with this model, computational analysis revealed that the electrostatic–hydrogen bond switch mechanism notably intensifies the affinity of PABDs for PA (Figure 1B) [22]. In agreement with these findings, it was observed both experimentally and by modeling simulations that divalent cations such as calcium can regulate the charge of PA [15,23].

## 5. Effect of Phosphatidic Acid Fatty Acyl Chain Composition

Mass spectrometry analysis showed that more than 40 different PA species are found in individual cell types [14,24], raising the possibility that these different forms of PA may have different biological functions. As a consequence, one could postulate that these different PA species display target specificity for the different PABDs. Probing this hypothesis, we recently tested in a liposome-binding assay the interaction of three PABDs, and found that the PABD of Spo20p, Opi1p, and PDE4A1 display a global predilection for long and unsaturated fatty acids [14]. Furthermore, although modest, preferences for the relative binding to different PA species were also observed among these PABDs. This was especially the case regarding the length of the sn-1 fatty acyl chain and the unsaturation status of the sn-2 chain [14]. Using molecular dynamics simulation, Spo20p–PABD was predicted to embrace an interfacial orientation displaying a large portion of hydrophobic amino acids embedded in the membrane, presumably interacting with fatty acids through hydrogen bonds [16]. One possible interpretation for differences found in PABD binding regarding unsaturation of PA may rely on the diverse composition in hydrophobic residues that vary from five to seven in the hydrophobic face of the three PABDs tested [14]. It must be pointed out that the number of positive charges also varies between PABDs, suggesting that the number of PA molecules that interact directly with one PABD is variable. This number can be as high as six for Spo20p (Figure 1D) [16]. As binding to PA by these short PABD polypeptides does not require a proper core-structured domain, it is more likely that hydrophobic insertion into membranes, together with direct ionic interaction between positively charged amino acids and more than a few PA molecules, is responsible. Thus, a strict specificity for each PA form is not to be expected, as is the case for the distinct phosphoinositides [25]. A similar organization was recently proposed for the PABD of the cell death-inducing DFF45-like effector A (CIDEA), which facilitates embedding into the phospholipid monolayer of lipid droplets through its ability to bind PA [26]. On the other hand, the PABD of GTPase dynamin-related protein 1 (Drp1) presents a clear preference for PA with saturated fatty acids, independently of their contribution to membrane curvature or lipid packing, suggesting a direct interaction of the PABD of Drp1 and fatty acids of PA [27,28].

## 6. Effect of pH on Phosphatidic Acid Binding

In a theoretical membrane bilayer model made of the zwitterionic lipid PC associated with a marginal amount of PA molecules, it was found that the degree of deprotonation of PA might be changed during protein binding, therefore improving the protein adsorption free energy. This phenomenon could be directly affected by pH. By investigating if the electrostatic–hydrogen bond switch process directly affects PA–PABD binding through non-electrostatic interactions via the hydrogen potential, the May group found that this was indeed the case [22]. From these pilot studies, it was postulated that the electrostatic–hydrogen bond switch participates in mechanisms that allow proteins to differentiate PA within a membrane containing higher amounts of other anionic lipids such as PS [22]. Despite many approximations in the model used, this work suggests the existence of an additional non-electrostatic contribution to binding, further increasing the sensitivity of this pH dependence. Consequently, the affinity of PABDs for membranes can drop dramatically after a physiological decrease in pH. In total agreement with modulation of PA–protein interactions by pH, as proposed by the electrostatic–hydrogen bond switch model, the Loewen group showed that the negative transcriptional regulator Opi1p regulates PA binding in a pH-sensitive manner, as a consequence of membrane biogenesis and general metabolism in yeast [29]. Starvation induces a drop in cytosolic pH in yeast and, consequently, Opi1p is released from the ER membrane and shuttles to the nucleus where it modulates gene expression, leading to membrane biogenesis shutdown. Conversely, in normal growth conditions where pH is neutral, Opi1p is restricted to the ER membrane, permitting membrane biogenesis. Thus, nutrient depletion effectively shuts down growth through a process that involves an on-and-off Opi1–PA dependent recruitment to the ER, in line with the idea that PA acts as a pH sensor. For the moment, there is no homologue of Opi1 in mammals, and it is not known if this pH regulation of protein–PA binding occurs for other PA targets. However, nuclear magnetic resonance and molecular dynamics simulations revealed that the PABD of the protein Dishevelled (Dvl) also binds PA in a pH-sensitive manner, therefore modulating its ability to interact with the Frizzled receptor and the plasma membrane [30].

## 7. Conclusions

This review highlights the current requirements for in-depth study of lipid–protein interactions, taking into consideration the comprehensive physical chemistry of individual membrane lipids, such as PA, in the context of a global membrane bilayer. The recent literature, based on experimental and computational evidence, supports the notion that the electrostatic–hydrogen bond switch in the phosphate headgroup of PA associated with positioning of the phosphate headgroup in close proximity to the hydrophobic interior of the membrane confers unique properties to PA, in comparison with other anionic membrane lipids. The data currently available establish that curvature stress and local lipid composition in the membrane, together with the particular biophysical properties of PA, are important factors in the binding characteristics of PABDs (Figure 1B,C). A minimalist theoretical model called the electrostatic–hydrogen bond switch model, largely based on the unique ionization properties of the phosphomonoester of PA, proposes that the initial membrane interaction of a cytosolic PABD is electrostatic in nature. This initial interaction could allow sampling of the local environment to differentiate PA from the other anionic lipids such as PS. After PA–protein interaction via hydrogen bonds between basic residues in binding domains, and potentially between hydrophobic residues and PA fatty acyl chains, PABDs change from a lightly bound to a docked state on the membrane. During this progression, the ionization of PA can switch from −1 to −2, further anchoring the PABD to the membrane [20]. Our own calculation estimated that the Kd of PA for the PABD of Spo20p, Opi1p, and PDE4A1 is in the micromolar range [14], in agreement with moderate affinity binding. However, the progressive α-helical organization, together with evidence from dynamic modelization suggesting that different PABDs could associate with more than one PA molecule, could significantly affect these numbers. It must be kept in mind that PA binding differentially affects protein activity. In some cases, PA increases membrane translocation or residency, whereas, in other cases, enzymatic activity could be directly modulated. These considerations reflect the large amount of work that lies ahead of us to fully understand the many biological functions of the simplest glycerophospholipid. Finally, binding of basic amino acids to the headgroup of PA, likely together with the probable contribution of hydrophobic forces and membrane geometry, provides an important mode of PA–protein interaction, as described in this review. However, this model is clearly not limited to amino acid–lipid interactions, as additional molecules such as small molecule inhibitors have also been shown to be effective for PA binding [31].
